# Disseminated tuberculosis in a child during the COVID-19 pandemic: a case report and literature review

**DOI:** 10.3389/fimmu.2023.1249878

**Published:** 2023-09-14

**Authors:** Taoping Weng, Yaqiong Dong, Niwen Huang, Chenqu Zhao, Lei Zhang, Shan Cao, Jing Tang, Danni Zhang, Xianming Zhang

**Affiliations:** Department of Respiratory and Critical Care Medicine, The Affiliated Hospital of Guizhou Medical University, Guiyang, China

**Keywords:** tuberculosis, disseminated tuberculosis, COVID-19, immunosuppression, treatment, case report

## Abstract

**Background:**

Disseminated tuberculosis is an uncommon but devastating form of tuberculosis, possibly developing with the immune response of patients. COVID-19 infection may produce an immunosuppressive effect with possible implications for tuberculosis dissemination.

**Case presentation:**

A 17-year-old female patient with a history of tuberculous pleurisy presented to the hospital with a high fever and life-threatening dyspnea after contracting a COVID-19 infection. Her condition deteriorated rapidly with grand mal epilepsy and acute gastrointestinal bleeding with a grossly depressed CD4 T-cell count, which was indicative of her profoundly immunosuppressed state. After identifying *Mycobacterium tuberculosis* in her cerebrospinal fluid and a subcutaneous abscess in her left lower back, she was diagnosed with disseminated tuberculosis involving both lungs, the central nervous system, the terminal ileum, the liver, bilateral adnexal tissue, and subcutaneous soft tissue in accordance with the chest and abdominal CT. Empirical treatment was initiated with dexamethasone (5 mg/day) and an anti-tuberculosis regimen of isoniazid, rifampicin, pyrazinamide, amikacin, and meropenem, which was replaced with faropenem after she left the hospital. The therapeutic effect was considered satisfied in the second month of follow-up.

**Conclusion:**

To the best of our knowledge, we report the first case report of disseminated tuberculosis after COVID-19 infection. Tuberculosis may disseminate and progress during the COVID-19 pandemic, requiring more significant studies to provide better diagnosis and treatment options for the co-infection.

## Introduction

Tuberculosis (TB) is an ancient communicable disease that is a major cause of ill health and one of the leading causes of death globally. There were approximately 10.6 million patients with TB in 2021, according to the Global Tuberculosis Report (1), with partial increases in 2021 due to the coronavirus (COVID-19) pandemic. This includes impacts on the provision of and access to essential TB services, the number of people diagnosed with TB and notified as TB cases through national disease surveillance systems, and the TB disease burden, including incidence and mortality. Furthermore, COVID-19 may lead to TB progression and fatal outcomes with the increased production of inflammation factors (2). Usually, the predilection sites of tuberculosis were the lungs, while over 15% of tuberculosis cases occur in the form of extrapulmonary infections that can affect any tissue in the body and are particularly difficult to diagnose and treat (3). Dissemination of *Mycobacterium tuberculosis* out of the lungs is thought to be more than just a rare event leading to extrapulmonary tuberculosis, but rather a prerequisite step that occurs during all infections, producing secondary lesions that can become latent or productive (4). COVID-19 may diminish the pool of *Mycobacterium tuberculosis*-specific memory T cell responses, with possible implications for TB disease progression (5) and dissemination. Here we report a case of the diagnosis and treatment of a child with disseminated tuberculosis during the epidemic period of COVID-19.

## Case presentation

A 17-year-old girl presented to our hospital on 31 January 2023, with 2 days of intermittent fever, dyspnea, and a maximum temperature of 38.5°C after a surgical operation for a perianal abscess. She had a history of tuberculous pleurisy, which was diagnosed the previous year, and complained of regular anti-tuberculosis treatment for the whole year. At the time of presentation, she was still on the anti-tuberculosis regimen of isoniazid, rifampicin, pyrazinamide, ethambutol, moxifloxacin, and linezolid. Meanwhile, the girl was infected with COVID-19, which was diagnosed by real-time PCR in December 2022. She denied a history of immunodeficiency virus (HIV) infection and hepatitis B and C. On 29 January 2023, she received an operation of incision and drainage of a perianal abscess at another hospital, and then the young patient experienced fever with chills, distress, and dyspnea. She came to our hospital for further emergency treatment.

**Figure 1:**
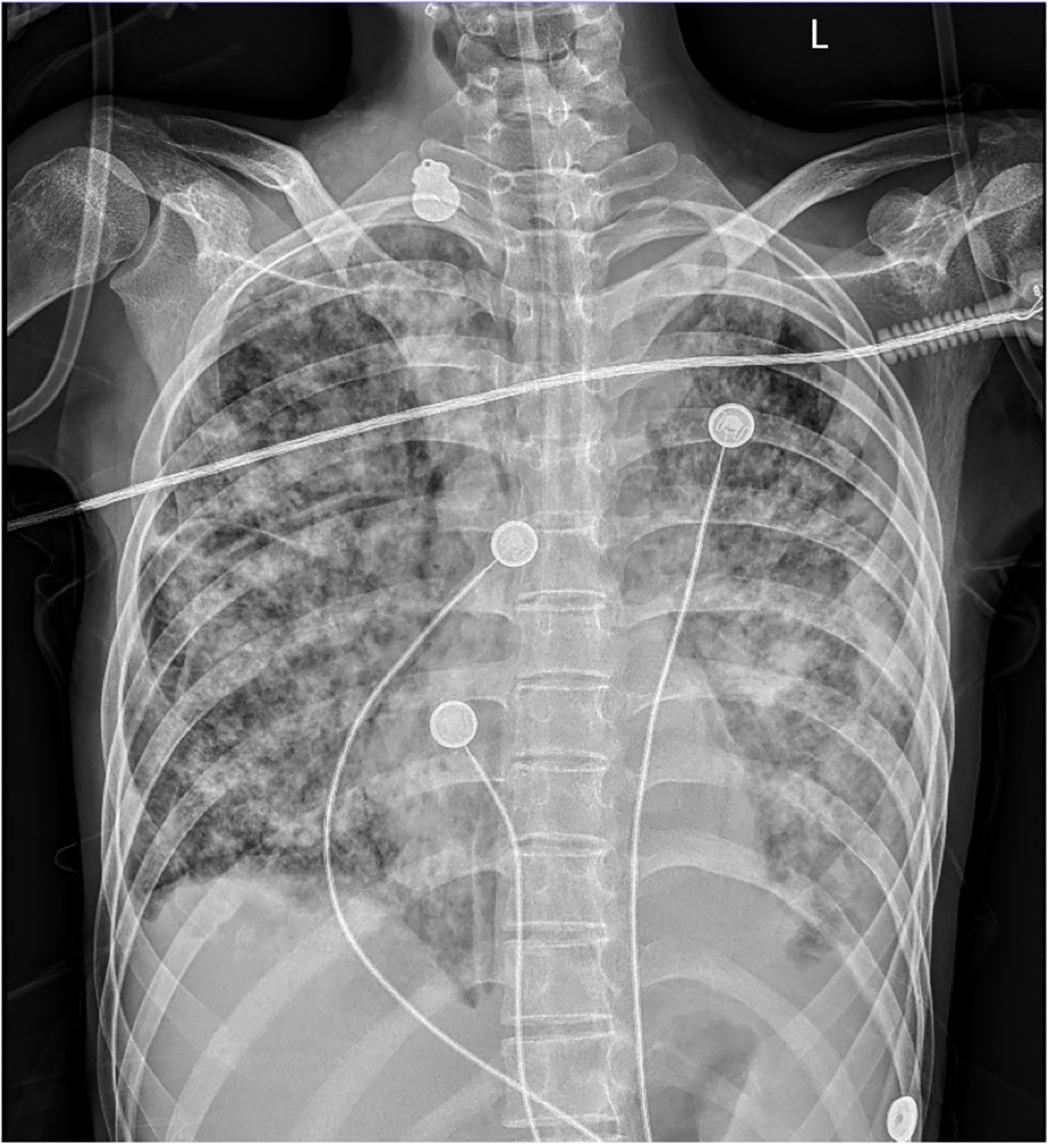
Bedside chest radiography. Diffuse infectious foci in both lungs with a slight bilateral pleural effusion.

On admission, she complained of obvious dyspnea with a temperature of 37.8°C, a heart rate of 167 per minute, a respiratory rate of 59 per minute, a blood pressure of 132/92 mmHg, and an oxygen saturation in room air of 74%. She was admitted to our respiratory intensive care unit due to critical hypoxia. Physical examination revealed moist rales in both lungs, slight neck rigidity, a positive Kerning’s sign, a positive Brudzinski’s sign, a regional subcutaneous mass with fluctuation in the left lower back, and a perianal wound after the operation. No other abnormal signs were found. Her laboratory results showed elevated white blood cells (15.91 g/L, normal value 4.1–11 g/L) with a mainly increasing count of neutrophils (14.34 g/L, normal value 1.8–8.3 g/L), anemia with decreased hemoglobin (86 g/L, normal value 114–145g/L), elevated C-reactive protein (89.98 mg/L, normal value < 5 mg/L), elevated serum adenosine deaminase (24.1 U/L, normal value < 20 U/L), and decreased CD4-T-lymphocytes (234/uL, normal value 554–1109/uL). The results of HIV infection and hepatitis B and C tests were negative. Considering intermittent fever, sputum smears and sputum and blood cultures of bacteria and fungus were also negative. Her bedside chest radiograph showed diffuse infectious foci in both lungs with a slight bilateral pleural effusion (**Figure 1**).

Unexpectedly, this patient soon developed grand mal epilepsy with loss of consciousness, a fixed gaze, and rigidity. We immediately operated on the lumbar puncture to obtain a specimen of her cerebrospinal fluid (CSF) to identify the cause of the epilepsy after the seizure was controlled. Her CSF results showed elevated interleukin-6 (421.70 pg/mL) with normal levels of CSF routine, biochemistry, and adenosine deaminase. Meanwhile, the CSF culture of bacteria was negative. Considering the possibility of tuberculous meningitis, an acid-fast bacilli smear, GeneXpert MTB/RIF, and next-generation sequencing (NGS) of her CSF were detected. Her CSF acid-fast bacilli smear and GeneXpert MTB/RIF were all negative. Simultaneously, her cranial computed tomography (CT) scan showed no abnormalities. Remarkably, *Mycobacterium tuberculosis* was detected according to the results of NGS, with evidence of high sensitivity and specificity in the diagnosis of tuberculous meningitis (6, 7). Tuberculous meningitis was confirmed, which led to the young patient’s epilepsy seizure. Combined with her tuberculous pleurisy history and the diffuse foci in both of her lungs, this girl was diagnosed with disseminated tuberculosis involving at least the lungs, pleura, and central system. Considering the girl had moderate anemia, we stopped the linezolid with its main adverse effect of myelosuppression and continued the anti-tuberculosis regimen of isoniazid, rifampicin, pyrazinamide, and ethambutol, accompanied by the antibiotic piperacillin/tazobactam, to control pneumonia diagnosed on the bedside radiography. Meanwhile, thymalfasin (thymosin-alpha 1) was also used as an immune system enhancer for the treatment of the immunosuppressed patient with a grossly depressed CD4 T-cell count. The child’s dyspnea subsided, her temperature sustained a normal level, and epilepsy never occurred again. The treatment seemed to be effective and the girl was transferred to the general ward of the respiratory department on 8 February 2023.

**Figure 2:**
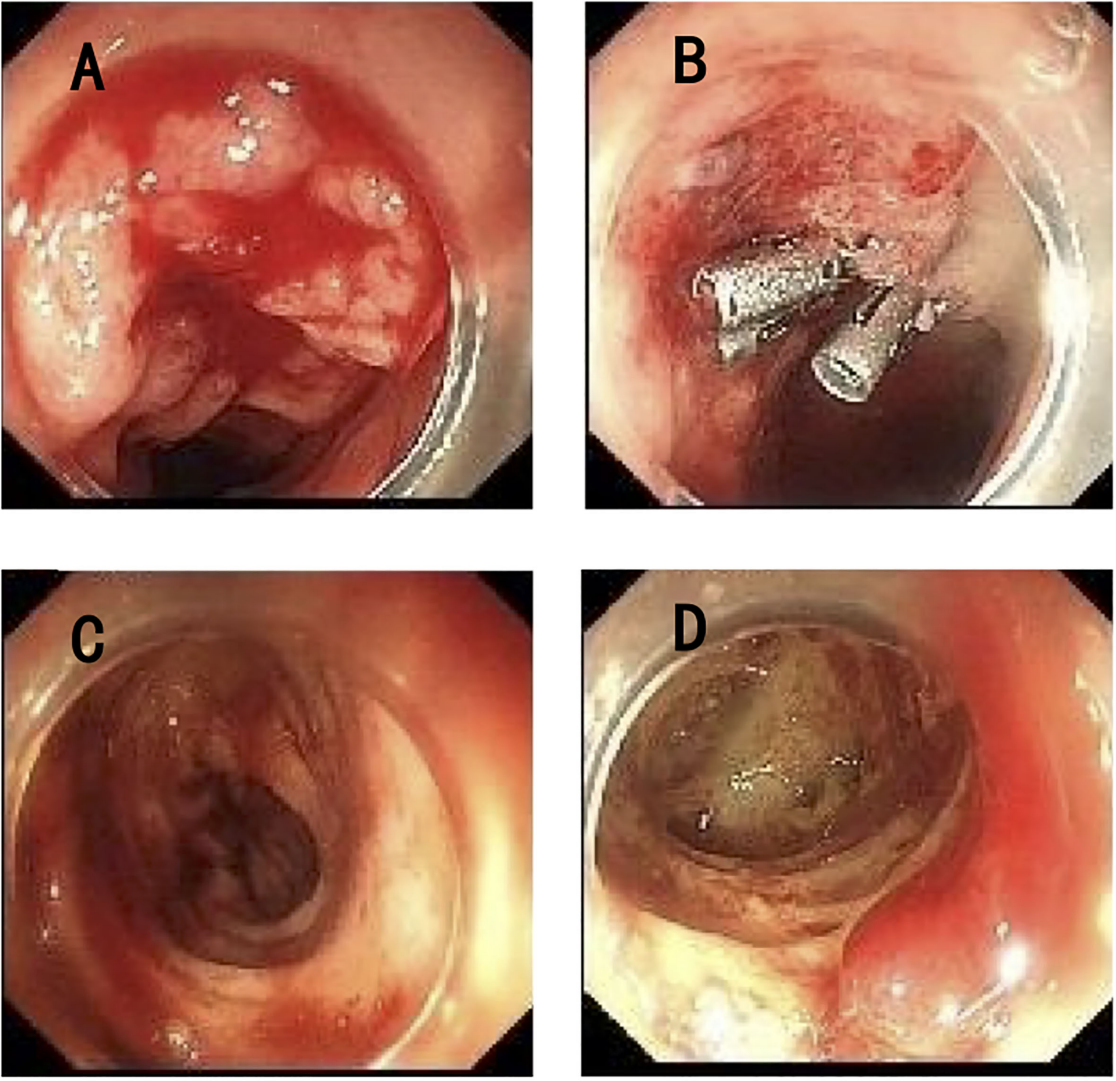
Endoscopy of the lower gastrointestinal tract. **(A)** Multiple ulcers with active bleeding in the terminal ileum. **(B)** Argon plasma coagulation and hemoclip therapy in the terminal ileum. **(C)** Multiple ulcers in the rectum. **(D)** Anal fistula.

Nevertheless, the patient developed acute gastrointestinal bleeding with black tarry stool and severe anemia of 50g/L hemoglobin on the second day in the general ward, and the child complained of obvious left hypogastralgia. A physical examination showed unstable vital signs with a heart rate of 144 per minute, a respiratory rate of 25 per minute, a blood pressure of 98/52 mmHg, and a severe anemic appearance. Immediately, the patient was transferred to the intensive care unit again for monitoring and stabilization. Though persistent internal hemostatic treatment was carried out, there was still active gastrointestinal bleeding. Therefore, a gastroscopy and colonoscopy were performed. There was no active bleeding on the gastroscopy, while the colonoscopy revealed multiple hemorrhagic ulcers in the terminal ileum (**Figure 2A**), as well as ulcers in the rectum (**Figure 2C**) and anal fistula (**Figure 2D**). Argon plasma coagulation and hemoclip therapy (**Figure 2B**) were performed so that the gastrointestinal bleeding was resolved with a significant improvement. Since there was active bleeding in the terminal ileum, we did not get a biopsy of the lesions, which made it difficult to make a differential diagnosis between intestinal tuberculosis and Crohn’s disease. We adjusted the treatment plan by adding the glucocorticoid (dexamethasone sodium phosphate, 5mg/day) for this girl with tuberculous meningitis, which was strongly recommended according to the World Health Organization (WHO) guidelines (8). In the meantime, the anti-tuberculosis regimen was regulated into isoniazid, rifampicin, pyrazinamide, amikacin (0.2g, I.V., q12 h), and meropenem (0.5g, I.V., q8 h), considering most bacteria and *Mycobacterium tuberculosis* were sensitive to the beta-lactam antibiotic, which was also recommended by the WHO guidelines for the treatment of drug-resistant tuberculosis (9).

**Figure 3:**
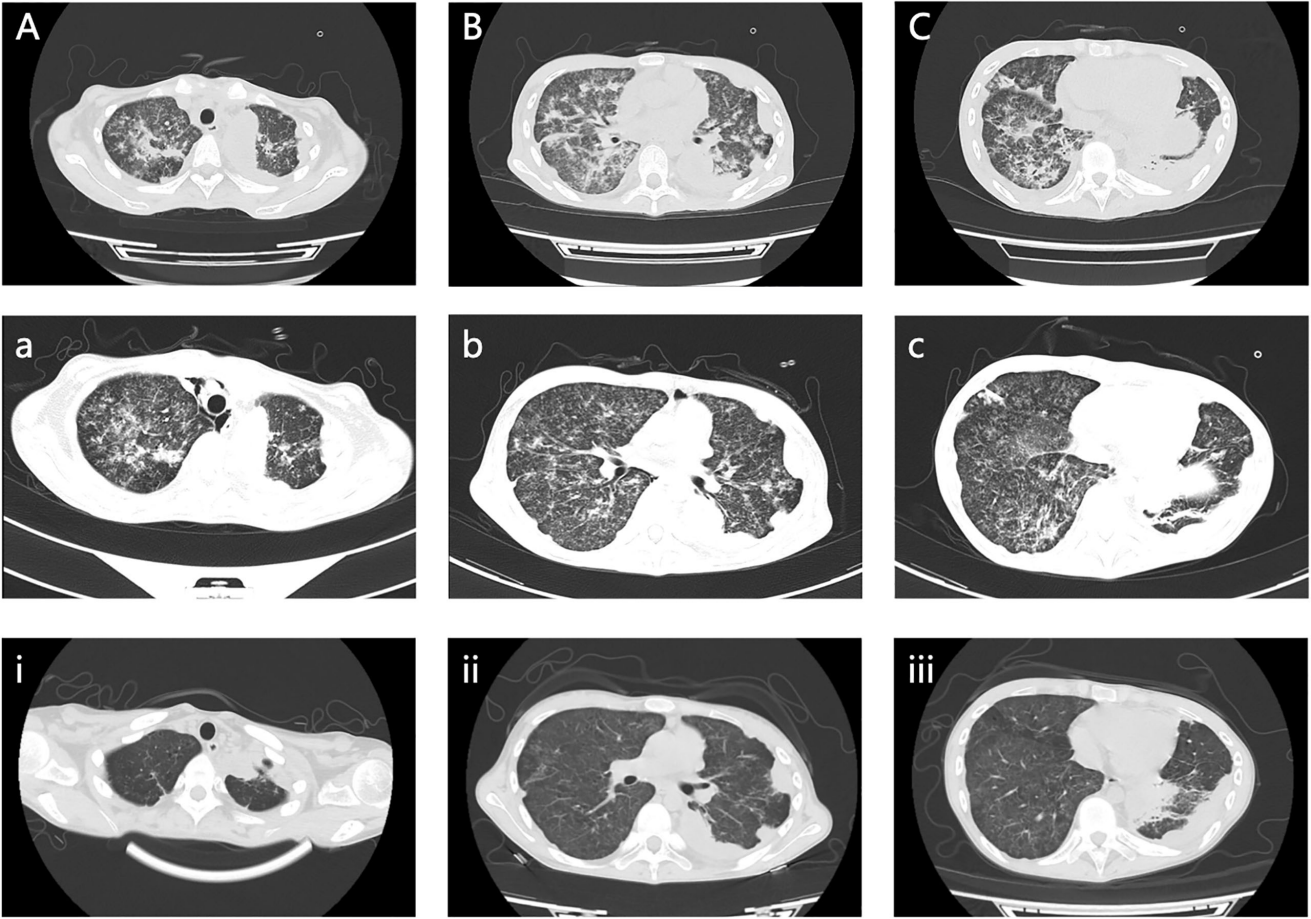
Chest and abdominal CT scan images. **(A–C)** Multiple infectious foci in both lungs and bilateral pleural effusion on 17 February 2023. **(a, b, c)** Multiple infection foci in both lungs were reduced on 6 March 2023. **(i, ii, iii)** Clearly decreased foci in both lungs on 28 April 2023.

**Figure 4:**
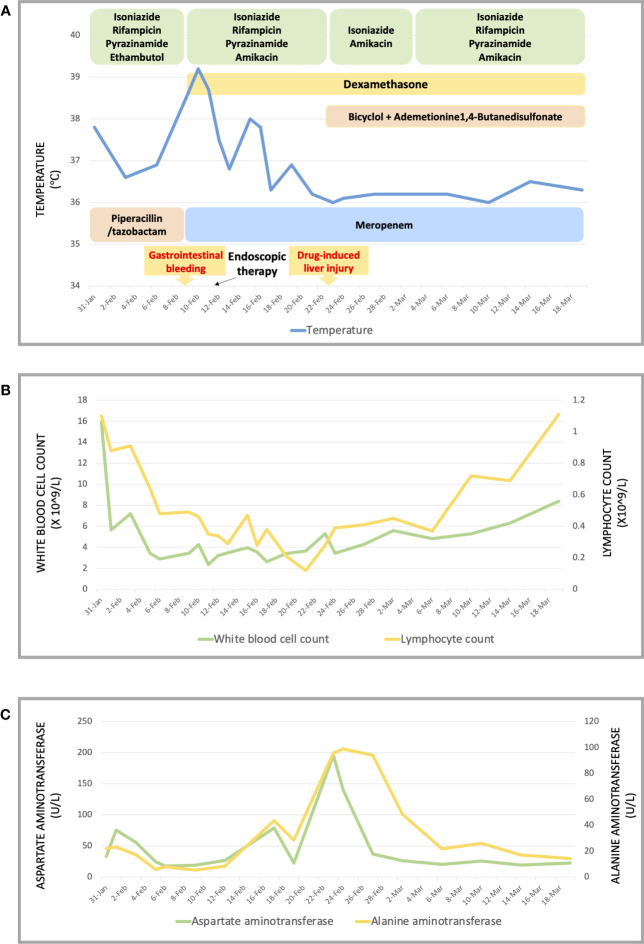
Treatment and observation during hospitalization. **(A)** Monitoring of body temperature and treatment. **(B)** Counting of white blood cells and lymphocytes. **(C)** Level of aspartate aminotransferase and alanine aminotransferase.

The symptoms apparently subsided after our therapy and nursing care, and her vital signs were maintained at a normal level. Consequently, the patient was transferred to the general ward, where she could undergo chest and abdominal CT scans after the life-threatening stage. Her chest and abdominal CT showed multiple infectious foci and miliary nodules, considering tuberculosis and bacterial pneumonia, bilateral pleural effusion (**Figures 3A–C**), a low-density lesion in the left lobe of the liver, suspecting hepatic abscess, and slightly high-density and gas-accumulated lesions in bilateral adnexal and subcutaneous encapsulated effusion in the left lower back, considering tuberculosis cold abscess. We considered that the multiple lesions at the different sites originated from disseminated tuberculosis. The girl then underwent percutaneous puncture drainage of the subcutaneous encapsulated abscess, and her pus specimen acid-fast bacilli smear was positive, which established the diagnosis of disseminated tuberculosis. Thus, the girl continued the anti-tuberculosis regimen of isoniazid, rifampicin, pyrazinamide, amikacin, and meropenem, along with dexamethasone. The patient responded well to the treatment, with marked improvement in fever and lymphopenia (**Figures 4A, B**). She also suffered from a drug-induced liver injury, which interrupted the anti-tuberculosis therapy. After receiving liver function-protecting treatment containing bicyclol and ademetionine, her transaminase recovered to a normal level (**Figures 4A, C**). She underwent chest and abdominal CT on 6 March 2023, and, encouragingly, the multiple infection foci in both lungs and subcutaneous effusion in the left lower back were reduced (**Figures 3a–c**). The patient was discharged on 23 March 2023, continuing with an oral anti-tuberculosis regimen of isoniazid (0.3 g/day), rifampicin (0.45 g/day), pyrazinamide (1.0 g/day), and faropenem (0.2 g, T.i.d.). Constructive follow-up is still ongoing, with an obvious absorption of lesions on bilateral lungs in the second month (**Figure 3i–iii**), and we will adjust the anti-tuberculosis regimen based on the therapeutic efficacy and adverse effects of the patient.

## Discussion and conclusion

Disseminated tuberculosis can cause systemic inflammatory response syndrome. Our report describes the case of a girl with tuberculous pleurisy under regular anti-tuberculosis therapy who developed disseminated tuberculosis involving both lungs, the central nervous system, the terminal ileum, the liver, bilateral adnexal tissue, and subcutaneous soft tissue after COVID-19 infection. The female patient’s grossly depressed CD4 T-cell count was indicative of her profoundly immunosuppressed state, closely related to the extrapulmonary disseminated tuberculosis (10). SARS-Cov2 will probably continue to circulate (11), making the diagnosis and treatment of disseminated tuberculosis in patients with COVID-19 more important for clinicians.

Individuals infected with *Mycobacterium tuberculosis* may either have a latent tuberculosis infection or develop active tuberculosis disease. For active tuberculosis disease, a small subset of patients (19.3–39.3%) present with either primary extrapulmonary tuberculosis or concurrent pulmonary involvement (12). Pathogen-associated molecular pattern signaling, antigen presentation, and immune recognition may mediate latency induction and pathogen reactivation, which are also believed to be important in establishing the site of disease presentation and dissemination (13). Additionally, the recovery of the immune response profile of inflammatory cytokines was observed in children with pulmonary tuberculosis but not in those with extrapulmonary tuberculosis after 6 months of treatment, which suggested the host immune response following treatment is specific to the disease rather than due to the within-host defense and cannot explain why one individual develops pulmonary tuberculosis while another develops extrapulmonary tuberculosis (14). Our patient developed disseminated tuberculosis after COVID-19 infection, which may be associated with abnormal immune responses.

Little evidence is available about how the co-infection of SARS-Cov2 and *Mycobacterium tuberculosis* impairs the host’s immune responses (15). Tuberculosis status may be associated with COVID-19 infection and exacerbation (16, 17), possibly because of the increased abundance of circulating myeloid subpopulations, which are also found in the lungs of patients with severe COVID-19 (18). Conversely, COVID-19 infection may play a role in active and latent tuberculosis. Increased IFN production and the type I and III IFN response signatures are significantly upregulated in COVID-19 (2), which may lead to tuberculosis progression and a fatal outcome. Moreover, many viruses, including SARS-CoV-2, cause a temporary immunosuppressive effect, which could lead to the reactivation and dissemination of *Mycobacterium tuberculosis* infection (19). A significant reduction in the frequency of *M. tuberculosis*-specific CD4 T-cells in patients with COVID-19 could affect the ability of the host to control latent or new *M. tuberculosis* infection (5, 20, 21). For our patient, other causes of immunosuppression were excluded, including chronic hepatitis B and C, HIV infection, primary immunodeficiency diseases, a depressed response to vaccination, and cancer. However, critical infection-induced immunosuppression (22) could not be differentiated without the result of *M. tuberculosis*-specific CD4 T-cells. Furthermore, longitudinal studies are required to investigate whether homeostatic reexpansion or peripheral redistribution of the *M. tuberculosis*-specific memory T cell pool has an impact on tuberculosis clinical outcomes after COVID-19 recovery.

The optimal anti-tuberculosis regimen for drug-susceptible tuberculosis usually consists of well-known first-line drugs, including rifampicin, isoniazid, pyrazinamide, and ethambutol. A meropenem/faropenem-containing regimen was designed for our patient with disseminated tuberculosis, despite no evidence of drug resistance. *M. tuberculosis* possesses a class C beta-lactamase that can inactivate carbapenems (23), while meropenem was the most stable carbapenem in the presence of the chromosomally encoded *blaC* beta-lactamase (24), considered a potentially useful anti-tuberculosis drug and classified by the WHO as Group C for drug-resistant tuberculosis (9). Meanwhile, oral faropenem is stable against the *blaC* enzyme present in *M. tuberculosis*, which managed to successfully kill *M. tuberculosis* growth *in vitro (* 25). The activity of carbapenems against mycobacteria has been extensively reported in the past several years (26), for instance, carbapenems can limit activity in a more physiologically hypoxic model emulating granuloma conditions (27). In particular, carbapenems occupy a significant niche in treating life-threatening infections (28). Our young patient received first-line drugs and fluoroquinolones for a whole year with a poor prognosis. Thereby, we made the meropenem/faropenem-containing regimen, which produced obvious therapeutic efficacy in the period of admission. Complementarily, ethambutol was not utilized considering the ocular toxicity, which may have generated confusion from the symptoms of epilepsy in this patient with tuberculous meningitis.

The process, from the initial symptoms of fever and dyspnea to the clinical diagnosis of disseminated tuberculosis, was tortuous but meaningful for us. Our young female patient is continuing the anti-tuberculosis therapy, and we will closely follow up on the effectiveness of her treatment and her prognosis.

To the best of our knowledge, ours is the first case report of disseminated tuberculosis after COVID-19 infection. Tuberculosis and other diseases may progress and develop with COVID-19 co-infection, yet studies related to tuberculosis and COVID-19 interaction are still limited. There is a need for more valuable research results to provide better diagnosis and treatment options for those patients.

## Data availability statement

The original contributions presented in the study are included in the article/**Supplementary Material**. Further inquiries can be directed to the corresponding author.

## Ethics statement

Written informed consent was obtained from the parents of the patient for the publication of this Case report and any accompanying images.

## Author contributions

TW, YD, and NH collected clinical data and conducted the literature review. TW wrote the manuscript. CZ, LZ, SC, JT, and DZ generated and interpreted data. All authors contributed to the revision of the manuscript for important intellectual content, approved the final version, and agreed to be accountable for all aspects of the work.
