# Supplementary material for: Disseminated tuberculosis in a child during the COVID-19 pandemic: a case report and literature review
Source: Front Immunol. 2023 Sep 14;14:1249878. doi: 10.3389/fimmu.2023.1249878 (PMC10536161; doi:10.3389/fimmu.2023.1249878)
Supplement: Supplementary file 1 [file DataSheet_1.pdf]

Follow-up Records of the Patient during Treatment

	Before Admitted	Presented to Hospital	In Hospital	Left Hospital	2 months after Discharged	4 months after Discharged
Disagnosis	Tuberculous Pleurisy	Unclear	Disseminated Tuberculosis involving both lungs, the Central Nervous System, the Terminal Ileum, the Liver, bilateral Adnexal Tissue, and Subcutaneous Soft Tissue			
Treatment Regimen	Anti-tuberculosis Regimen (Isoniazide, Rifampicin, Pyrazinamide, Ethambutol, Moxifloxacin, and Linezolid)	Anti-tuberculosis Regimen (Isoniazide, Rifampicin, Pyrazinamide, Ethambutol) and Piperacillin/tazobactam	Anti-tuberculosis Regimen (Isoniazide, Rifampicin, Pyrazinamide, Amikacin, and Meropenem) and Thymalfasin, Dexamethasone, Bicyclol and Ademetionine	Anti-tuberculosis Regimen (Isoniazide, Rifampicin, Pyrazinamide, and Faropenem)		
Chest CT/X-ray	N/A	Diffuse Infectious Foci in both Lungs	Multiple Foci and Miliary Nodules	Multiple Foci Reduced Obviusly	Obvious Absorption of Lesions	N/A
Hepatic Fuction	N/A	Normal Level	Drug-induced Liver Injury	Normal Level	Normal Level	Normal Level
Epilepsy	N/A	Suffered		No		
Gastrointestinal Bleeding	N/A	Suffered		No		
CD4 T-cell count	N/A	234/uL	N/A	456/uL	N/A	N/A
